# Clinicopathological analysis and prognostic treatment study of angiosarcoma of the breast: a SEER population-based analysis

**DOI:** 10.1186/s12957-023-03030-9

**Published:** 2023-05-09

**Authors:** Lizhi Teng, Shuai Yan, Juntong Du, Ru Yang, Peng Xu, Weiyang Tao

**Affiliations:** 1 Department of Breast Surgery, The First Affiliated Hospital of Harbin Medical University, Harbin, 150001 China; 2 Key Laboratory of Acoustic, Optical and Electromagnetic Diagnosis and Treatment of Cardiovascular Diseases, Heilongjiang, China; 3 The Cell Transplantation Key Laboratory of National Health Commission, Harbin, Heilongjiang, 150001 China; 4 Key Laboratory of Hepatosplenic Surgery, Ministry of Education, Harbin, Heilongjiang, 150001 China

**Keywords:** Breast, Angiosarcoma, SEER, Treatment, Surgery

## Abstract

**Introduction:**

Breast angiosarcoma is a rare malignancy of endovascular origin, accounting for less than 1% of all mammary cancers. Our aim was to explore clinicopathological features and the factors associated with prognosis.

**Methods:**

We extracted information from the Surveillance, Epidemiology, and End Results Program (SEER) for all patients with breast angiosarcoma between 2004 and 2015. Chi-square analysis was used to compare the clinicopathological features in all patients. Overall survival (OS) was assessed using the Kaplan and Meier method. Univariate and multivariate analyses were performed to evaluate the factors associated with prognosis.

**Results:**

A total of 247 patients were included in the analyses. The median OS of patients with primary breast angiosarcoma (PBSA) and secondary breast angiosarcoma (SBAB) was 38 months and 42 months, respectively. The 1-, 3- and 5-year OS with PBSA was 80%, 39%, and 25%, respectively, and the 1-, 3- and 5-year OS with SBAB was 80%, 42%, and 34%, respectively. Multivariate analysis revealed that tumor size (*p* = 0.001), tumor grade (*p* < 0.001), tumor extension (*p* = 0.015), and tumor spread (*p* < 0.001) were statistically significant factors for OS. Partial mastectomy with radiation (HR = 0.160, 95% CI, 0.036–0.719, *p* = 0.016), partial mastectomy with chemotherapy (HR = 0.105, 95% CI, 0.011–1.015, *p* = 0.052), and partial mastectomy (HR = 0.125, 95% CI, 0.028–0.583, *p* = 0.007) were related to significantly better OS outcomes in primary angiosarcoma.

**Conclusion:**

Primary breast angiosarcoma has a better clinical phenotype than secondary breast angiosarcoma. Although overall survival was not statistically significant, primary breast angiosarcoma was better than secondary breast angiosarcoma with systemic therapy. Depending on the outcome of survival, partial mastectomy is effective in treating primary breast angiosarcoma.

## Introduction

Breast angiosarcoma is a rare malignancy of endovascular origin and a subtype of soft-tissue sarcoma, accounting for less than 1% of all breast malignancies and less than 5% of all sarcomas [1, 2]. Clinically, breast angiosarcoma is divided into two categories: primary breast angiosarcoma (PBAS) and secondary breast angiosarcoma (SBAS). PBAS frequently arises from the breast gland, breast soft tissue, subcutis, or dermis; it is difficult to distinguish from benign disease and usually occurs in young women. It often presents clinically as painless or palpable injury and is sometimes accompanied by a change in the color of the skin, such as skin depigmentation [3]. However, SBAS is always related to two aetiological factors: radiotherapy after breast-conserving mastectomy, in which the latency period for onset is usually less than 10 years; and ipsilateral limb lymphedema called Stewart-Treves syndrome (STS). SBAS usually occurs in older women; the tumor develops in the skin tissue and may invade the parenchyma of the mammary gland, and it is sometimes associated with erythaematous plaques or nodules [4, 5].

Given the rarity of this disease, many institutions have inadequate samples to research. The majority of relevant studies have examined the clinicopathological features of PBSA, but few studies have explored SBSA and the treatment of breast angiosarcoma. Additionally, many of these articles are small case reviews and case reports, and we cannot derive definitive clinical features that affect prognosis. A study from The University of Texas M. D. Anderson Cancer Center examined of 55 patients with angiosarcoma to determine its clinical features and prognosis, and only SBAB was found to occur predominantly in young patients receiving radiotherapy, but there was no difference in OS between PBSA and SBAB [6].

Clinically, there is occult pathogenesis of angiosarcoma, and there is nonspecific imaging on ultrasound and mammography, posing a great challenge to its diagnosis. Treatment in the sarcoma service is currently recommended for the management of breast angiosarcoma [7]. In past research, some authors have thought that multidisciplinary combination therapy including surgery, radiation, and systemic chemotherapy can improve survival in patients [8]; however, due to the small sample size, there are inherent limitations in the experimental results. A review has shown that there is no clear evidence of specific treatments for specific subtypes of angiosarcoma and mostly symptomatic treatment, and the inability to treat specifically for a particular type may also be one of the reasons for the worse OS of breast angiosarcoma [9]. Similarly, in multivariate analysis, it was found that radiotherapy and systematic chemotherapy do not significantly prolong the overall survival of patients in other studies.

Based on the current research status, we chose to use the Surveillance, Epidemiology, and End Results Program (SEER) database to extract and analyze data on PBSA and SBAB. The main objective of this study was to explore the clinical characteristics and treatment decisions of PBSA and SBAB to solve the remaining treatment problems, and to improve the survival of patients.

## Methods

This study was performed with approval from the Surveillance, Epidemiology, and End Results Program (SEER) database. The SEER database is a publicly available, comprehensive database of demographic information for selected US states and counties (approximately 35% of the US population), which compiles information on prevalence, morbidity, mortality, and survival rates (http://seer.cancer.gov/about/overview.html). We obtained permission from SEER to access the study data (reference number 14492-Nov2020). Given that the study is based on a strict registration process, the need for informed consent was waived. In addition, the study was exempted from Institutional Review Board approval, as the use of SEER data does not enable the identification of patient information.

We retrospectively reviewed the information we collected and finally determined that 394 patients had angiosarcoma who received treatment between 2004 and 2015 due to the limitation of the years of tumor size and tumor extension inclusion in the database. Studies have divided breast angiosarcoma into PBSA and SBAB, and the determination of PBSA and SBAB was based on the criteria of Saira et al. [10] and Taimur et al. [11]. The inclusion criteria were as follows: (1) the patient had only one primary angiosarcoma or multiple malignancies, but angiosarcoma was the first diagnosis of PBSA, and (2) the patient had a non-first-stage malignancy that had previous breast cancer, lung cancer, or soft tissue cancer in the chest wall as SBAB. The exclusion criteria were as follows: (1) patients with previous cancer that was not related to the breast or chest wall but a malignancy in other parts, such as brain cancer, skin cancer, and bone cancer, were excluded, and (2) patients with incomplete clinical information on tumor size, stage, and grade were excluded. Ultimately, we identified 247 patients for inclusion in this study for further analysis.

The clinical characteristics of the follow-up patients were reviewed: ICD-O-3 histology codes as haemangiosarcoma (9120), race, tumor onset location, tumor grade, tumor size, tumor stage, depth, number of primary tumors, chemotherapy, radiation, and type of initial surgery. Classify tumors at the nipple in the central group of the breast. The return of the SEER database divides the pathology grading into Grade I, Grade II, Grade III, and Grade x. Based on the maximum dimension of the tumor, we divided it as ≤ 5, 5–10, or > 10 cm. The historic tumor stage used was local, regional, and distant. In the tumor extension group, tumors that were confined to breast tissue and fat were defined as confined, tumors that were infiltration of lymph nodes around the tumor and skin involvement were defined as local infiltration, and tumors that were attachment or fixation to pectoral muscle or underlying tissue were defined as deep. To describe specific surgeries in the database, we divide the surgeries into partial mastectomy, mastectomy, and unknown. Unknown refers to patients who have not undergone surgery or who have refused surgical treatment. Patients who have received radiation therapy in the past are unknown about the dose of radiation therapy, and the specific regimen of chemotherapy and the cycle of chemotherapy are also unknown.

For the 2 patient groups, the clinical characteristics of patients are mainly realized as frequencies and percentages as categorical variables. Overall survival (OS) was defined from the initial angiosarcoma diagnosis to the time of death. OS was assessed by the Kaplan‒Meier method, and the differences between the different groups were compared using the log-rank test. The median of the results is included in the table as an estimate. Chi-square testing was applied to compare distributions between groups. Univariate analysis was performed for PBSA and SBAB prognostic variables with the log-rank test and Cox regression. Variables that were significant in univariate analysis were incorporated into multivariate Cox proportional hazards regression analysis. The extractor of the data was SEER 18, and the statistical analysis was performed with SPSS 22. *P* values < 0.05 or less were considered significant.

## Results

During the period 2004–2015, on account of the CS being size limited, 394 patients were included as identifying angiosarcoma of the breast from the SEER database. All the patients underwent prior malignancy history or radiation history, and 148 patients were removed because they lacked some data associated with clinical characteristics. On the basis of the inclusion criteria, 247 patients were included for further analyses.

### Description of the clinical characteristics of PBSA and SBAB

A total of 247 patients were identified. One hundred patients (40.5%) had PBSA, including 1 male patient and 246 female patients, and 147 patients (59.5%) had SBAB, all of whom were female. The clinical characteristics are described in Table 1. In our cases, patients diagnosed with SBAB (median age 50–54 range 15–85 +) were 20 years younger than patients diagnosed with PBSA (median 70–74 range 25–85 +). The majority of tumors (67.0 vs. 65.3%) occurred in the overlap or entire breast. At diagnosis, 169 patients were more likely to be poorly differentiated (68.4%); of these, 118 were secondary angiosarcoma (80.3%), but 78 patients were moderately or highly differentiated (31.6%). In addition, 74 patients presented with locally advanced stage in PBSA (74.0%), while 74 patients presented with regionally advanced stage in SBAB (50.3%); both patients presented with distant metastasis. There were 75 patients in the primary group for one stove (75.0%); nevertheless, none of the patients had a total number of sites in the PBSA. In 79 patients, tumor extension was confined to breast tissue in the PBSA (79.0%); in 79 patients, it exhibited local infiltration or a deep muscle layer in SBAB (53.7%). Analysis of radiation showed that more patients were inclined to accept radiation treatment in PBSA, but few patients followed in SBAB (31.0 vs. 17.0%). The most frequent predisposing conditions were surgeries (96.0 vs. 99.3%). In summary, the results of the present study suggested that PBSA has a better clinical phenotype and that the patient is more likely to receive treatment than SBAB.

**Table 1:** Comparison of patient clinical characteristics

	**Primary angiosarcoma, *n* (%)**	**Secondary angiosarcoma, *n* (%)**	***p* value**
Total	100	147	
Median age	50–54	70–74	
< 65	64 (64.0)	38 (25.9)	
≥ 65	36 (36.0)	109 (74.1)	*p* < 0.001
Race			
White	79 (79.0)	133 (90.5)	
Non-white^a^	21 (21.0)	14 (9.5)	*p* = 0.011
Site			
Central^b^	4 (4.0)	13 (8.9)	
Inner quandrant	13 (13.0)	14 (9.5)	
Outer quandrant	16 (16.0)	24 (16.3)	
Overlap	31 (31.0)	26 (17.7)	
NOS^c^	36 (36.0)	70 (47.6)	*p* = 0.058
Grade			
G1	21 (21.0)	10 (6.8)	
G2	28 (28.0)	19 (12.9)	
G3	27 (27.0)	47 (32.0)	
Gx^d^	24 (24.0)	71 (48.3)	*p* < 0.001
Tumour size			
< 5 cm	49 (49.0)	79 (53.8)	
5-10 cm	33 (33.0)	54 (36.7)	
> 10 cm	18 (18.0)	14 (9.5)	*p* = 0.15
Tumour spread			
Local	74 (74.0)	67 (45.6)	
Regional	18 (18.0)	74 (50.3)	
Distant	8 (8.0)	6 (4.1)	*p* < 0.001
Tumour extension			
Confined	79 (79.0)	68 (46.3)	
Local infiltration	10 (10.0)	41 (27.9)	
Deep	11 (11.0)	38 (25.8)	*p* < 0.001
Total number			
1	75 (75.0)	0 (0)	
2	21 (21.0)	96 (65.3)	
≥ 3	4 (4.0)	51 (34.7)	*p* < 0.001
Chemotherapy			
Yes	37 (37.0)	32 (21.8)	
No	63 (63.0)	115 (78.2)	*p* = 0.09
Radiation			
Yes	31 (31.0)	25 (17.0)	
No	69 (69.0)	122 (83.0)	*p* = 0.01
Surgery			
Yes	96 (96.0)	146 (99.3)	
No	4 (4.0)	1 (0.7)	*p* < 0.001

^a^We recoded detailed race information into four major categories, the non-White population includes Black, American Indian/Alaska Native, and Asian Pacific Islander

^b^Classify tumor at the nipple in the central group of the breast

^c^Entire breast: 3/4 or more of breast involved with tumor

^d^Undifferentiated, anaplastic, and grade IV

### Comparison of the survival and prognosis of PBSA and SBAB

**Fig. 1:**
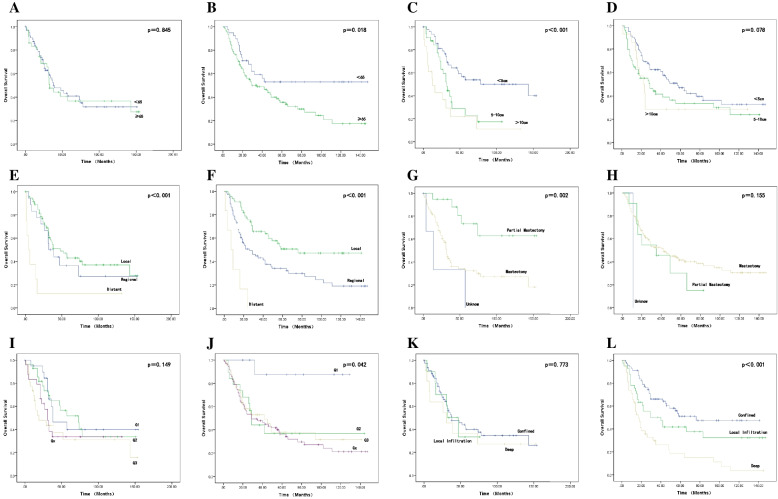
The Kaplan–Meier survival curves on the impact of univariate analysis on OS. In PBSA and SBAB, **A** and **B**, for age comparison. **C** and **D**, for tumor size comparison. **E** and **F**, for tumor spread comparison. **G** and **H**, for surgery comparison. **I** and **J**, for grade comparison. **K** and **L**, for tumor extension comparison

On univariate analysis (Table 2 and Fig. 1), in the primary patient subgroup, age, tumor site, tumor grade, number of primary sites, chemotherapy, radiation, and tumor extension did not reach statistical significance to predict OS outcomes. Prognostic markers of tumor size, tumor stage, and surgery were significant variables. Patients who had tumor sizes less than 5 cm were obviously better than patients who had larger tumors (*p* < 0.001). Patients who received partial mastectomy had better OS outcomes than patients who refused surgery or accepted mastectomy (*p* = 0.002). Advanced tumor spread was associated with a tendency towards OS outcomes in the primary cohort (*p* < 0.001). In multivariate analysis (Table 3) of OS that included tumor size, tumor stage, and tumor extension, only tumor size was demonstrated to be statistically significant (HR 1.856 95%:1.304–2.641, *p* = 0.001).

**Table 2:** Univariate analysis of overall survival in patients with PBSA and SBAB

	**Primary angiosarcoma**			**Secondary angiosarcoma**		
	**Median OS, mo**	**95%CI**	***p* value**	**Median OS, mo**	**95%CI**	***p* value**
Median age						
< 65	39	20.779–57.221		NA	NA	
≥ 65	33	23.505–42.495	*p* = 0.845	35	18.651–51.349	*p* = 0.018
Race						
White	36	23.705–48.295		42	29.668–54.332	
Non-white	39	11.243–66.757	*p* = 0.763	19	13.500–24.500	*p* = 0.456
Site						
Central	31	0.594–61.406		48	0–114.644	
Inner quadrant	NA	NA		22	0–62.564	
Outer quadrant	52	21.747–79.253		NA	NA	
Overlap	31	17.658–44.342		29	16.868–41.132	
NOS	34	14.474–53.526	*p* = 0.303	29	13.545–44.455	*p* = 0.151
Grade						
G1	38	12.245–63.755		NA	NA	
G2	73	39.724–106.276		29	25.277–32.723	
G3	19	0–40.336		42	16.790–67.210	
Gx	31	23.793–38.207	*p* = 0.149	29	9.757–48.243	*p* = 0.042
Tumor size						
< 5 cm	143	24.180–261.820		57	36.842–77.158	
5–10 cm	32	24.558–39.443		29	12.806–45.194	
> 10 cm	13	5.296–20.704	*p* < 0.001	22	12.833–31.167	*p* = 0.078
Tumor spread						
Regional	32	16.347–47.653		26	13.191–38.809	
Localized	48	28.395–67.605		76	NA	
Distant	4	0–9.544	*p* < 0.001	7	0–14.201	*p* < 0.001
Tumor extension						
Confined	38	19.424–56.576		76	NA	
Local infiltration	32	13.406–50.544		40	17.095–62.905	
Deep	31	0–64.447	*p* = 0.773	17	11.887–22.113	*p* < 0.001
Total number						
1–2	37	24.285–49.715		42	27.111–56.889	
≥ 3	38	NA	*p* = 0.303	29	1.867–56.133	*p* = 0.527
Chemotherapy						
Yes	37	26.769–47.231		29	11.371–46.629	
No	38	24.961–51.039	*p* = 0.650	45	29.575–60.425	*p* = 0.737
Radiation						
Yes	39	5.274–72.726		35	3.630–66.370	
No	36	21.980–50.020	*p* = 0.284	45	29.076–60.924	*p* = 0.684
Surgery						
Unknow	14	0–30.003		11	NA	
Partial mastectomy	NA	NA		35	5.868–64.132	
Mastectomy	32	28.273–35.727	*p* = 0.002	42	27.504–56.496	*p* = 0.155

**Table 3:** Multivariate analysis of overall survival in patients with PBSA and SBAB

	**Primary angiosarcoma**			**Secondary angiosarcoma**		
	**HR**	**95%CI**	***p* value**	**HR**	**95%CI**	***p* value**
Median age	—			1.538	0.891–2.655	*p* = 0.122
Grade	—			2.118	1.570–2.858	*p* < 0.001
Tumor size	1.856	1.304–2.641	*p* = 0.001	—		
Tumor spread	1.382	0.833–2.293	*p* = 0.211	1.687	1.109–2.567	*p* = 0.015
Tumor extension	—			2.118	1.570–2.858	*p* < 0.001
Surgery	1.357	0.705–2.612	*p* = 0.361	—		

In the secondary patient subgroup, however, on univariate analysis (Table 2 and Fig. 1), tumor site, tumor size, number of primary sites, chemotherapy, radiation, and surgery did not achieve statistical significance for OS outcomes. OS outcomes were significantly worse in patients above 60 years of age (*p* = 0.018). Patients who had deep tumor location or local infiltration fared prominently worse than did patients presenting with confined breast tissue (*p* < 0.001). Compared with the controls, tumor grades were closely related to OS, and low grade (G1) had better OS outcomes (*p* = 0.042). Patients with local spread had improved OS outcomes compared with those with regional spread or distant spread (*p* < 0.001). In multivariate analysis (Table 3), poor differentiation, deep tumor extension and distant spread were independently correlated with worse OS outcomes, with hazard ratios of 2.118 (95% CI 1.570–2.858, *p* < 0.001), 1.687 (95% CI 1.109–2.567, *p* = 0.015), and 2.118 (95% CI 1.570–2.858, *p* < 0.001), respectively.

We then assessed the influence of the pathological data included in Table 1 on the OS outcomes of PBSA and SBAB. The median OS with PBSA or SBAB was 38 months vs. 42 months (95% CI 23.001–52.999 vs. 95% CI 28.893–55.107). The 1-, 3- and 5-year OS rates for PBSA were 80%, 39%, and 25%, respectively, and the 1-, 3- and 5-year OS rates for SBAB were 80%, 42%, and 34%, respectively.

### PBSA and SBAB therapeutic effects

Despite soft sarcomas originating from other parts of the body with a high probability of recurrence, the mainstay of therapeutic modalities has been surgical excision (Table 1). Of the 100 PBSA patients, 96.0% were treated with surgery, 37.0% with chemotherapy, and 31.0% with radiation. Among the 147 SBAB patients, 99.3% received surgery, 21.8% received chemotherapy, and 17.0% received radiation. As shown in Tables 2 and 3, surgical treatment alone is statistically meaningful in patients with PBSA, and there was no statistically significant difference when we considered only the influences of chemotherapy or radiation on long-term survival.

However, angiosarcoma has a poor prognosis, and a multidisciplinary combination therapy has been proposed to improve survival in the clinic. However, since SEER collection is limited, and we cannot browse for detailed chemotherapy and radiation information. Instead, we can investigate the effects of surgery combined with adjuvant treatments on survival outcomes with limited treatment information, and we discussed different combined treatment modalities. As shown in Table 4, in the PBSA patient group, univariate analysis showed that partial mastectomy (HR = 0.160, 95% CI, 0.036–0.719, *p* = 0.017) and partial mastectomy with radiation (HR = 0.061, 95% CI, 0.006–0.587, *p* = 0.016) were related to significantly better OS outcomes than mastectomy and mastectomy with radiation in the surgery combined radiation subgroup. In the surgery combined with chemotherapy subgroup, we also demonstrated that partial mastectomy with chemotherapy (HR = 0.105, 95% CI, 0.011–1.015, *p* = 0.052) and partial mastectomy (HR = 0.125, 95% CI, 0.028–0.583,* p* = 0.007) were associated with better OS outcomes. However, in SBAB patients, surgery combined with adjuvant treatments was not correlated with improved OS outcomes.

**Table 4:** Univariate Cox analysis of combined treatment in patients with primary angiosarcoma and secondary angiosarcoma

	**Primary angiosarcoma**			**Secondary angiosarcoma**		
	**HR**	**95%CI**	***p* value**	**HR**	**95%CI**	***p* value**
Surgery combined radiation						
Unknown	1			1		
Partial mastectomy + radiation	0.061	0.006–0.587	0.016	0.210	0.021–2.057	0.210
Partial mastectomy	0.160	0.036–0.719	0.017	0.316	0.036–2.759	0.316
Mastectomy + radiation	0.440	0.125–1.551	0.202	0.228	0.029–1.793	0.228
Mastectomy	0.486	0.149–1.586	0.232	0.192	0.026–1.419	0.192
Surgery combined chemotherapy						
Unknow	1			1		
Partial mastectomy + chemotherapy	0.105	0.011–1.015	0.052	0.509	0.052–4.959	0.509
Partial mastectomy	0.125	0.028–0.583	0.007	0.205	0.023–1.793	0.205
Mastectomy + chemotherapy	0.412	0.120–1.416	0.159	0.186	0.024–1.439	0.186
Mastectomy	0.511	0.156–1.677	0.268	0.198	0.027–1.463	0.198

## Discussion

Breast angiosarcoma is a rare disease in the clinic, and we divided it into PBSA and SBAB types based on similar studies in previous literature [10, 11]. PBSA accounts for less than 1% of breast cancer cases, with an incidence of 4.5 cases per million, but the incidence of SBAB was reported to be less than 0.5% [12]. Due to the rarity of the disease, it causes great challenges in the diagnosis, prognosis, and treatment of the disease. This study sought to determine the correlation between PBSA and SBAB by comparing 100 primary patients from the SEER database with 147 secondary patients, as well as to explore the diagnosis and treatment decisions of the disease. Our results suggested that the size of the tumor, the stage of the tumor, and the depth of the tumor in breast angiosarcoma often mark a progressive tumor phenotype. The size of the tumor in PBSA led to a worse prognosis, and the grading of the tumor in the SBAB context represents a worse prognosis with the depth of the tumor. However, the 1-, 3-, and 5-year OS of the two groups of patients were compared, and no significant difference was found. Under different monotherapy and combination therapy models, we found that simple surgical treatment, partial mastectomy combined with radiation and partial mastectomy combined with chemotherapy in PBSA can improve OS outcomes, but different treatments in SBAB were not statistically significant.

In a review of past research, the incidence of angiosarcoma in recent years has gradually increased from 1.52 to 2.04 per year, and breast cancer patients account for a relatively high proportion [13]. At present, the pathogenesis of PBSA is unclear, but SBAB is usually associated with postoperative edema, lymphatic blockade, perioperative infection, and radiation therapy. The onset of the disease is usually 4 to 7 years after breast-conserving therapy. PBSA usually presents clinically as a palpable mass, while SBAB usually presents with cutaneous changes such as abrasions, violaceous skin rash, and red papules or pain and discomfort in deep tissues, and the presentation is polycentric [14]. These clinical manifestations are similar to the manifestations of many benign tumors, and it is difficult for us to diagnose them through these manifestations, which also leads to our inability to diagnose and treat them through early clinical manifestations, resulting in delays in the condition and poor prognosis. On the other hand, due to the polycentric nature of SBAB, we were also unable to accurately measure the size of the tumor and the edge of the tumor in the clinic. Therefore, it was difficult for us to achieve R0 resection clinically, which also led to the risk of local recurrence and metastasis in patients in disguise, and some patients may have the possibility of reoperation.

In terms of the grouping definition of tumor size, our classification divides it into < 5, 5–10, or > 10 cm groups based on previous literature (*p* = 0.15). Because tumors smaller than 5 cm in primary patients are the most reliable prognostic criterion for prognosis, the size of the tumor is closely related to the prognosis of the primary patients [15, 16]. This is consistent with the results of this study in univariate analysis and multivariate analysis. However, Keila [17] found that tumor size with 10 cm as the defining point was also prognostically correlated in secondary patients in univariate analysis and multivariate analysis. Through the analysis of the data, it was found that the tumor size in this study was 9.5 cm as the cutoff point, which was statistically significant (*p* < 0.001). However, from the perspective of clinical and collected data, tumors larger than 9.5 cm have exceeded the scope of the breast, the number of patients with large tumors in the clinic is few, and the prognosis is relatively worse; thus, the results obtained herein indicate only a “nominally” poor prognosis, and we did not use these data.

Our study revealed that the OS of angiosarcoma is poor and is not sensitive to various treatments, especially among patients with SBAB. The tumor itself grew quickly and was prone to distant metastasis, and early diagnosis and intervention are particularly important. However, traditional imaging had a certain degree of invisibility, such as mass under the mammograms, asymmetrical density, parenchymal disorders, skin thickening and microcalcification, and under ultrasound, and they were mostly manifested as masses and thickening of the skin, which are similar to many benign diseases. In the literature on Sona A, it is shown that MRI is diagnostically specific, manifested by low T2-weighted imaging in the rapidly enhancing part of the dermis and parenchyma lesions and T1-weighted imaging characterized by a fast initial and delayed washout, which can determine the extent of the tumor [18]. Therefore, it is recommended that patients with suspected angiosarcoma can use MRI for localization to determine the extent of the tumor, and at the same time, if the patient has a history of breast cancer radiation therapy, skin changes should be observed carefully, and if necessary, puncture biopsy should be performed to confirm the diagnosis.

There are also differences in prognosis due to the difference in PBSA and SBAB performance. In primary patients, in a review article of 42 cases of PBSA, the most significant prognostic factor was the size and grade of the tumor, while no difference in prognosis for radiotherapy-induced angiosarcoma was found [3]. This conclusion is similar to a previous article. In a review of 57 cases, the overall survival rate at 10 years was 62%, and pathological grade and tumor size were found to be associated with prognosis in univariate analysis, while multivariate analysis found that only pathological grade was associated with prognosis [19]. In another study of 16 cases of PBSA, the authors found that all patients underwent surgery with curative intent, and the overall survival rate was 13.6 months. The univariate analysis suggested that high-grade tumors predicted a worse prognosis, but tumor metastases had no effect on prognosis [20]. However, in our study, consistent with previous studies, the 1-, 3-, and 5-year OS rates for primary angiosarcoma were 80%, 39%, and 25%, respectively. Univariate analysis found that tumor size, tumor spread, and surgery were associated with prognosis, and multivariate analysis found that tumor size was independently associated with prognosis. Our research has demonstrated that in patients with PBSA, early diagnosis (when the tumor < 5 cm) and surgery can effectively improve the prognosis and survival of patients. In secondary patients, in a review article of 112 cases of SBAB, high-grade tumor patients (OS: 36 months) had a worse prognosis than lower-grade tumor patients (OS: 48 months) and a 5-year overall survival rate of 50.5% [6]. In addition, a review of the Jeffrey [21] article in 176 secondary patients found that OS at 3 years was 74%, and it was proven that positive surgical margins, tumor depth, and high grade were associated with prognosis. However, in another article review of the George [22], we found that age, radical resection, and margins were associated with prognosis in univariate and multivariate analyses, while tumor grading was not statistically significant. In our study, the 1-, 3-, and 5-year OS rates for secondary angiosarcoma were 80%, 42%, and 34%, respectively. Tumor grade, age, tumor spread, and tumor depth were found to be associated with prognosis in univariate analyses, and tumor grade, tumor stage, and tumor depth were associated with prognosis in multivariate analysis, consistent with previous studies. In this study, it was found that there were obvious differences in prognostic factors between PBSA and SBAB, but no significant differences were found in OS outcomes; thus, it was not recommended to manage the clinical and prognostic management of primary and secondary patients as usual. Among primary and secondary patients, SBAB is not related to tumor size; perhaps, it was not accurate in the measurement range due to its multicentric characteristics affecting the assessment of prognosis. Age is associated with prognosis, but we should consider comorbidities in older patients. It is possible that other comorbidities cause the OS of the patient to decline. Secondary patients are associated with the depth of the tumor in prognosis, possibly because the patient received radiation therapy in the past, resulting in an increased risk of developing disease in all areas of radiation therapy.

At present, there is still great controversy over the treatment of angiosarcoma. In a review article of 9 patients among secondary patients, surgery combined with radiotherapy improved survival, but chemotherapy did not have a survival benefit. Surgery to R0 in primary and secondary patients improves long-term survival and can reduce the risk of recurrence [19]. However, there were also articles reviewing the treatment of 28 patients, 15 of whom underwent axillary dissection, and suggested that chemotherapy was more inclined to use high-grade or lesions greater than 9.5 cm. A total of 22 patients were included in the review of Joshua [10], and neoadjuvant chemotherapy was found to have a survival benefit and demonstrated that multidisciplinary combination therapy could prolong long-term survival in patients. A review of the treatment of 16 patients in the Qun-Chao Hu [20] found that partial resection and total resection had no effect on survival in primary patients, and it was proven that patients who did not reach the R0 edge after surgery were only associated with postoperative recurrence and had no effect on survival. In secondary patients, the authors reviewed 176 patients in whom chemotherapy was used less frequently (41 vs. 4%) than other types of sarcomas and radical resection improved survival, but radiation therapy was not beneficial [21]. The results of this study are not consistent with those of previous studies. We only found in univariate analysis that surgical treatment was significant for primary patients, and the other variables were not statistically significant. Therefore, our analysis of the mode of combined therapy found that in primary patients, partial resection, partial resection combined with chemotherapy and partial resection combined with radiotherapy are all statistically significant in the treatment of primary patients. Although it was not statistically significant in the secondary group, we cannot deny the therapeutic significance of total resection combined with related adjuvant therapy clinically. Based on the results of this study, we believe that in primary patients, in surgery combined with chemotherapy and surgery combined radiotherapy, the HR of partial resection combined chemotherapy is 0.016, while the total resection combined chemotherapy HR is 0.440, and the partial resection combined with radiotherapy HR is 0.105, while the total resection combined with radiotherapy HR is 0.412. The reason for the occurrence of this treatment may be related to the size of the tumor. In patients with tumors smaller than 5 cm, most patients will choose partial resection, and the prognosis of patients with tumors < 5 cm is better than that of patients with tumors ≥ 5 cm, which may also indirectly affect the patient’s treatment outcome.

Although this study is the largest collection of risk factors to date, there are still data limitations. First, it is well established that radiotherapy is associated with SBAB, and information on the dose, extent, duration, and period of radiotherapy plays a crucial role in the evaluation of secondary angiosarcoma; however, this information was not available in the database. It has been shown that chronic lymphedema is an important risk factor for the development of angiosarcoma of the breast (STS), and fibrosis of the surrounding skin tissue caused by radiotherapy may be a factor in its development [23]. It is evident from previous cases that STS has a worse prognosis than other skin malignancies, and early diagnosis and treatment are imperative; however, STS is insidious, and many diagnoses rely on subjective assessment [24, 25]. In recent years, studies have been performed using TDC and indocyanine green (ICG) lymphography to objectively assess the status of lymph node circulation, providing new ideas for early diagnosis of the disease [26, 27]. Unfortunately, information on lymph node edema is not available in the SEER database.

## Conclusion

Primary breast angiosarcoma has a better clinical phenotype than secondary breast angiosarcoma. Through univariate and multivariate analyses, it was found that the prognosis of breast angiosarcoma is related to many clinical factors. Although overall survival was not statistically significant, primary breast angiosarcoma was better than secondary breast angiosarcoma with systemic therapy. In patients with secondary angiosarcoma, there was no statistically significant difference between treatment and survival in the current study, and new treatment modalities need to be further studied. Depending on the outcome of survival, partial mastectomy is effective in treating primary breast angiosarcoma.

## Data Availability

The datasets generated during and/or analyzed during the current study are available in the SEER database repository, Surveillance, Epidemiology, and End Results Program (cancer.gov).
